# In vitro behaviour of human gingival fibroblasts cultured on 3D-printed titanium alloy with hydrogenated TiO_2_ nanotubes

**DOI:** 10.1007/s10856-022-06649-4

**Published:** 2022-03-02

**Authors:** Yatong Guo, Xin Wang, Caiyun Wang, Su Chen

**Affiliations:** Multidisciplinary Treatment Center, Capital Medical University School of Stomatology, Beijing Stomatological Hospital, Beijing, 100050 China

## Abstract

Selective laser melting (SLM), as one of the most common 3D-printed technologies, can form personalized implants, which after further surface modification can obtain excellent osseointegration. To study the surface properties of SLM titanium alloy (Ti6Al4V) with hydrogenated titanium dioxide (TiO_2_)nanotubes (TNTs) and its influence on the biological behaviour of human gingival fibroblasts (HGFs), we used SLM to prepare 3D-printed titanium alloy samples (3D-Ti), which were electrochemically anodizing to fabricate 3D-TNTs and then further hydrogenated at high temperature to obtain 3D-H_2_-TNTs. Polished cast titanium alloy (MP-Ti) was used as the control group. The surface morphology, hydrophilicity and roughness of MP-Ti, 3D-Ti, 3D-TNTs and 3D-H_2_-TNTs were measured and analysed by scanning electron microscopy (SEM), contact angle metre, surface roughness measuring instrument and atomic force microscope, respectively. HGFs were cultured on the four groups of samples, and the cell morphology was observed by SEM. Fluorescence staining (DAPI) was used to observe the number of adhered cell nuclei, while a cell counting kit (CCK-8) was used to detect the early adhesion and proliferation of HGFs. Fluorescence quantitative real time polymerase chain reaction (RT–qPCR) and enzyme-linked immunosorbent assay (ELISA) were used to detect the expression of adhesion-related genes and fibronectin (FN), respectively. The results of this in vitro comparison study indicated that electrochemical anodic oxidation and high-temperature hydrogenation can form a superhydrophilic micro-nano composite morphology on the surface of SLM titanium alloy, which can promote both the early adhesion and proliferation of human gingival fibroblasts and improve the expression of cell adhesion-related genes and fibronectin.

**Figure:**
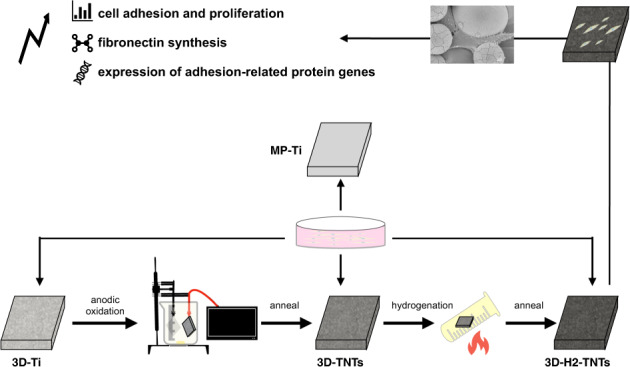
Graphical abstract

Graphical abstract

## Introduction

Implant restoration has become the choice of an increasing number of patients to repair missing teeth with the development of dental implant technology. Although standardized titanium implants and abutments are commonly used in the clinic with superior biocompatibility, they lack an individualized design and have difficulty satisfying the needs of some complicated cases. Therefore, the emergence of 3D printing technology provides reliable technical support for the manufacture and wide use of individualized implants [1]. With the aid of computer design, 3D printing technology can prototype implants rapidly and accurately while achieving better controllability. Selective laser melting (SLM) is one of the most common 3D printing additive manufacturing technologies. The titanium alloy produced by SLM not only shows good mechanical properties but also has the characteristics of a low elastic modulus and corrosion resistance [2].

A number of in vivo and in vitro studies have confirmed that SLM titanium alloys have good osteogenic properties [3, 4]. However, few studies have explored the effect of SLM titanium alloys on the formation of soft tissue, which constitutes the transgingival area around the implants. As the main cells of the connective tissue, the early adhesion and proliferation of HGFs integrated with the surface of implant material plays an important role in shaping the soft tissue cuffs, resisting the invasion of bacteria and preventing the epithelial root from migrating to ensure the long-term stability of osseointegration with the implant [5–7]. Studies have shown that the biological behaviour of cells is closely related to the surface morphology, roughness and hydrophilicity of the materials [8–10].

Previous studies by our team on modifying smooth titanium by preparing TiO_2_ nanotubes showed that TiO_2_ nanotubes could load drugs and that the formation of the biological coating had antibacterial and osteoconductive activity [11]. In addition, TiO_2_ nanotubes treated by high-temperature hydrogenation can promote the early adhesion and proliferation of osteoblasts and human gingival fibroblasts [12, 13] and the synthesis of extracellular matrix due to superhydrophilicity. However, the effect of preparing hydrogenated TiO_2_ nanotubes on 3D-printed titanium alloys on the biological behaviour of HGFs remains to be explored.

In this work, titanium alloy samples with microspherical structures were fabricated by a selective laser melting technique, nanotubes were formed on the samples by electrochemical anodic oxidation, and then the materials were hydrogenated at high temperature. We analysed the surface properties of the samples and evaluated their influence on adhesion and proliferation of human gingival fibroblasts, as well as the expression of adhesion-related proteins and genes. We speculated that human gingival fibroblasts could adhere rapidly and proliferate on the surface of the 3D-H_2_-TNTs. If the material is applied to the surface design of implant neck or healing abutment, it may be beneficial to form the rapid and good soft tissue sealing in the transgingival area at the early stage after implantation.

## Material and methods

### Sample preparation and treatment

**Fig. 1:**
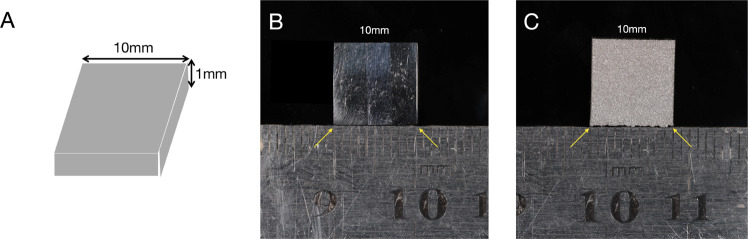
(**A**) Design sketch of samples (**B**) MP-Ti sample (**C**) 3D-Ti sample

As shown in Fig. 1(B), smooth titanium alloy samples (MP-Ti) produced as square titanium pieces with side length of 10 mm and thickness of 1 mm used as the control group were fabricated by lost wax process. We first made the wax moulds, which were cast by high-temperature wax loss to get the corresponding titanium alloy samples, and then after polishing with 2000 mesh sand paper to get the MP-Ti.

The 3D-Ti samples were manufactured by SLM with commercial grade V titanium powders (Ti6Al4V) with an average diameter of approximately 30 μm. The SLM processing parameters were adjusted to a laser power of 200 W, maximum laser scanning velocity of 5 m/s and laser spot size of 50 µm. As shown in Fig. 1(C), the samples in size of 10 × 10 × 1 mm^3^ were without other treatments after removing the support. They were successively ultrasonically cleaned with acetone, ethanol and deionized water and dried at room temperature before use.

The 3D-Ti plate was set as the anode, and the platinum plate was set as the cathode. They were immersed in ethylene glycol electrolyte containing 0.5 wt % NH_4_F and 10 vol % deionized water for 15 min with a 50 V constant voltage DC power supply during the process of anodic oxidation. Then, ethanol and deionized water were used to wash the 3D-Ti plate for 1 min, and the plate was dried in air. Subsequently, the samples were placed in a tubular furnace and annealed in air at 500°C for 2 h to produce 3D-TNTs. Some of the 3D-TNTs samples were sealed into a quartz tube filled with 0.95 × 10^5^ Pa hydrogen and placed in a tubular furnace to anneal at 500 °C for 4 h to prepare 3D-H_2_-TNTs.

The 3D-Ti, 3D-TNTs and 3D-H_2_-TNTs were used as the test groups. The samples used for cell culture were all treated with high temperature and high pressure sterilization before cell experiments.

### Sample characterization

Scanning electron microscopy (SEM, S-4800, Hitachi, Japan) served as the facility for the surface morphology observation of the samples. Atomic force microscopy (AFM, Nanoscope V, Veeco, USA) and a precision roughness profiler (JB-4C, Taiming Shanghai, China) were used to quantitatively analyse the surface roughness of MP-Ti and the other three SLM sample groups, respectively. We used simple random sampling method to directly draw three samples from each group and three different 5 µm^2^ areas were randomly measured on the surface of each sample. The average ± standard deviation (μm) data of each group was calculated, which was used to denote the surface roughness of the sample. An optical contact angle measuring device (OCA 15pro, Dataphysics, Germany) was used to measure the water contact angle of the samples and the simple random sampling method was also applied to select samples.

### Cell culture

Commercially available HGFs (HGF-1, CRL-2014; ATCC, Manassas, VA, USA) were first cultured in 10 ml Minimum Essential Medium *α* (*α*-MEM; Thermo Fisher Scientific, USA) containing 10 vol % foetal bovine serum (FBS; Thermo Fisher Scientific, USA) and 1 vol % antibiotics (penicillin/streptomycin, Thermo Fisher Scientific, USA) in the cell culture dish with a diameter of 10 cm at 37 °C under a 5% CO_2_ atmosphere in the CO_2_ incubator (BB150, Thermo Fisher Scientific, USA). We changed the medium every 3 days and passaged the cells by trypsin digestion when they grew 80% of the bottom of the dish. HGFs at passages 3–5 were used for the experiments.

### Biocompatibility study

The samples used for cell experiments were all in size of 10 × 10 × 1 mm^3^.

#### Cell morphology

Scanning electron microscopy (SEM, S-4800, Hitachi, Japan) served as the facility for cell morphology observation. HGFs were cultured on the surfaces of four groups of samples in 24-well plates at a density of 1 × 10^4^ cells/well. After cultivating for 1 h, 4 h and 1 day under standard conditions, the cells were fixed with 2.5% glutaraldehyde at 4 °C overnight, dehydrated with ethanol at different concentrations (50%, 75%, 90%, 95% and 100%), completely dehydrated in hexamethyldisilane (HMDS) solution and dried in a ventilator. Finally, the samples were observed after gold spraying.

#### Cell adhesion and proliferation assays

Fluorescence microscopy (IX73, OLYMPUS, Japan) was used to observe the DAPI (Beyotime Biotechnology, China) -stained early adhered nuclei, while Spectramax Paradigm (Molecular Devices, USA) was used to measure the absorbance of HGFs adhered on the surface of each group of samples, which had been treated with Cell Counting Kit-8 assay (CCK-8, Dojindo, Japan) to evaluate the early adhesion of cells. HGFs were inoculated on the surface of the samples in 24-well plates at a density of 5 × 10^4^ cells/well. Three samples were selected randomly from each group and then put in one 24-well plate. The samples were washed with phosphate-buffered saline (PBS, Thermo Fisher Scientific, USA) three times to remove unadhered cells after culturing for 0.5 h, 1 h and 4 h. Then, a few drops of DAPI were added to some of the samples, and the surface was covered for 10 minutes. Next, the dye was flushed away with running water, and the samples were blotted with filter paper. Finally, the samples were transferred to the microslide, and a drop of fluorescent encapsulation solution was added for observation. The other samples were transferred to a new 24-well plate, and 500 μL *α*-MEM of 10 vol % CCK-8 solution was added to each well. After incubating for 2 h under standard conditions, the absorbance values (ODs) were read at 450 nm by Spectramax Paradigm. A cell counting kit assay (CCK-8; Dojindo, Japan) was used to measure cell proliferation after culturing for 1 day, 3 days, 5 days and 7 days as described above.

#### Fibronectin synthesis by ELISA

A human fibronectin (FN) enzyme-linked immunosorbent assay kit (ELISA, Thermo Fisher, USA) was used to determine the fibronectin synthesis of HGFs. HGFs were cultured on each sample in a 24-well plate at a density of 2 × 10^6^ cells/well for 4 h and 1 day under standard conditions. At each time point, cell supernatant in each well of different groups was collected and analysed according to the ELISA protocol. Then, the OD value of the supernatant was read at 450 nm by the Spectramax paradigm, and the value was substituted into the standard curve equation to quantitatively calculate the concentration of FN.

#### Gene expression by RT–qPCR

The expression of adhesion-related protein genes and extracellular matrix protein genes were detected by RT–qPCR. Five samples were randomly selected from each group and placed in one well of a 6-well plate, and HGFs were added at a density of 2 × 10^6^ cells/well to each well. After culturing for 4 h and 1 day under standard conditions, total RNA was extracted from the adhered cells by a single-step method with TRIzol reagent (Invitrogen, USA). Then, cDNA was obtained by reverse transcription using the PrimeScript RTReagent Kit (Takara, Japan) in the light of the instruction. The primers (Shenggong Shanghai, China) commercially synthesized of focal adhesion kinase(FAK), integrin-β1(ITG-β1) and vinculin(VCL) were used as the template for the amplification of corresponding mRNA products, and all mRNA values were normalized according to GAPDH expression. An RT-qPCR system (CWBio, China) was used to analyse the expression of genes.

### Material toxicity test

In this section, five groups were set up, including 3D-TNTs, 3D-TNT_1/2_, 3D-TNT_1_, 3D-TNT_2_ and 3D-TNT_5._ The preparation process for 3D-TNTs was consistent with the method previously introduced. The anodized titanium samples were ultrasonically rinsed in ethanol and deionized water for 30 s, 1 min, 2 min and 5 min, respectively. After drying in air, the samples were placed in a tubular furnace for annealing treatment, which was heated to 500 °C for 2 h in an air environment, held for 2 h, and then cooled to room temperature with the furnace. After high-temperature and high-pressure sterilization, the five groups of materials were placed in a 24-well plate, and then 1 mL HGF cell suspension was added at a density of 5 × 10^4^ cells/ml to each well. Cell Counting Kit-8 assays (CCK-8, Dojindo, Japan) were used to detect the adhesion and proliferation of cells after culture for 4 h and 1 day as described previously.

### Intracellular reactive oxygen species detection

The 4 h group: Cell suspension was prepared by in situ loading probes into HGFs in accordance with the instructions of Reactive Oxygen Species Assay Kit (ROS, Beyotime Biotechnology, China). The Mp-Ti, 3D-Ti, 3D-TNTs and 3D-H_2_-TNTs samples after high-temperature and high-pressure sterilization were placed in a 24-well plate, and the blank and Rosup were set as the negative and positive control groups, respectively. The cell suspension was added to the corresponding wells with a blank in a 24-well plate. In the positive control group, Rosup was added according to the instructions after adding the cell suspension into the well. A 500 μL cell suspension was added to each well at a density of 5 × 10^5^ cells/ml and cultured for 4 h under standard conditions.

The 1d group:The Mp-ti, 3D-Ti, 3D-TNTs and 3D-H_2_-TNTs samples after high-temperature and high-pressure sterilization were placed in a 24-well plate. The set and treatment of the negative control group and positive control group were the same as the set and treatment of the negative control group and positive control group of the 4 h group. A 500 μL cell suspension was added to each well at a density of 5 × 10^5^ cells/ml and cultured for 1 day under standard conditions. The probe was loaded into the HGFs that adhered to the bottom of the plate and the surface of the samples according to the instructions of the Reactive Oxygen Species Assay Kit.

At 4 h and 1 day, we rejected the medium in each well of the Blank group and the Rosup group and added trypsin (Gibco, USA) to digest the cells. At the same time, the samples of each group were transferred to a new 6-well plate, and trypsin was added to the surface of the samples for digestion. The digestive juice of each well was transferred to a centrifugal tube and centrifuged at 1100 rpm for 5 min. Then, the supernatant was overwelled, and the cells were resuspended in 200 μL *α*-MEM medium in a new tube. The fluorescence intensity of each group was examined by flow cytometry (FCM, Accuri C6, BD, USA) under an excitation wavelength of 488 nm and an emission wavelength of 525 nm. Three parallel samples were set at each time point in each group.

## Statistical analysis

Experimental data are expressed as the mean ± standard deviation. At least three samples were tested in each group for material tests, while at least three parallel samples were set in each group and repeat the same operation at least twice at each time point for cell experiments. One-way ANOVA with SPSS 23.0 software and LSD tests were used to analyse the material surface roughness, water contact angle and cytological results. The result was statistically significant when the *P* value was less than 0.05 (*p* < 0.05).

## Results

### Sample characterization

**Fig. 2:**
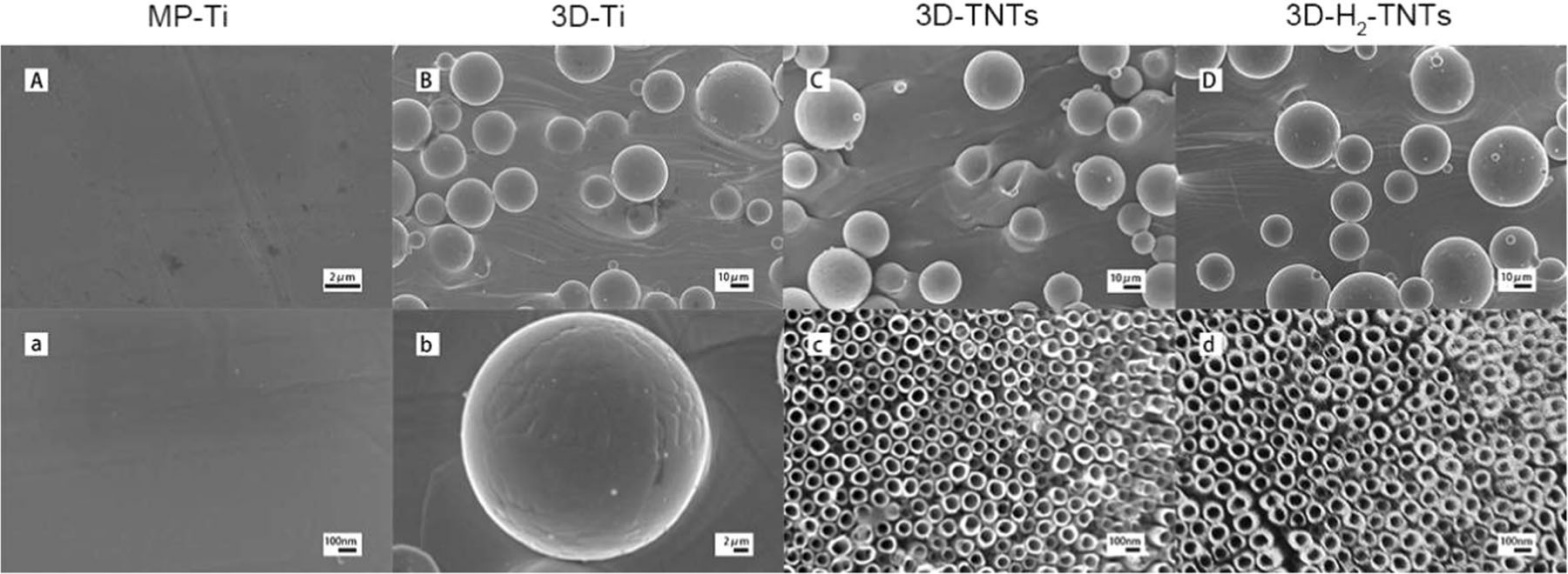
Sample characterization. SEM images of (**A**) the MP-Ti substrate at low magnification (×5000), (**a**) the MP-Ti substrate at high magnification (×50,000); (**B**) the 3D-Ti at low magnification (×500), (**b**) the 3D-Ti at high magnification (×2000); (**C**) the 3D-TNTs at low magnification (×500), (**c**) the 3D-TNTs at high magnification (×50,000); (**D**) the 3D-H2-TNTs at low magnification (×500), (**d**) the 3D-H2-TNTs at high magnification (×50,000)

The surface morphologies of the four groups of samples are shown in Fig. 2. Scanning electron microscopy showed that the surface of MP-Ti is flat and smooth, while there are a large number of incompletely melted titanium spheres with different sizes on the surface of the 3D-Ti substrate, and the diameter of most titanium spheres is in the range of 30–50 μm. Regular nanotubes with a diameter of approximately 80–100 nm protruded radially on the surface of the titanium spheres and perpendicular to the substrate were formed after anodic oxidation in the 3D-TNTs group at high magnification. In addition, some scattered microcrack structures could be observed between the nanotubes. There was no significant difference in surface morphology between 3D-TNTs and 3D-H_2_-TNTs.

**Fig. 3:**
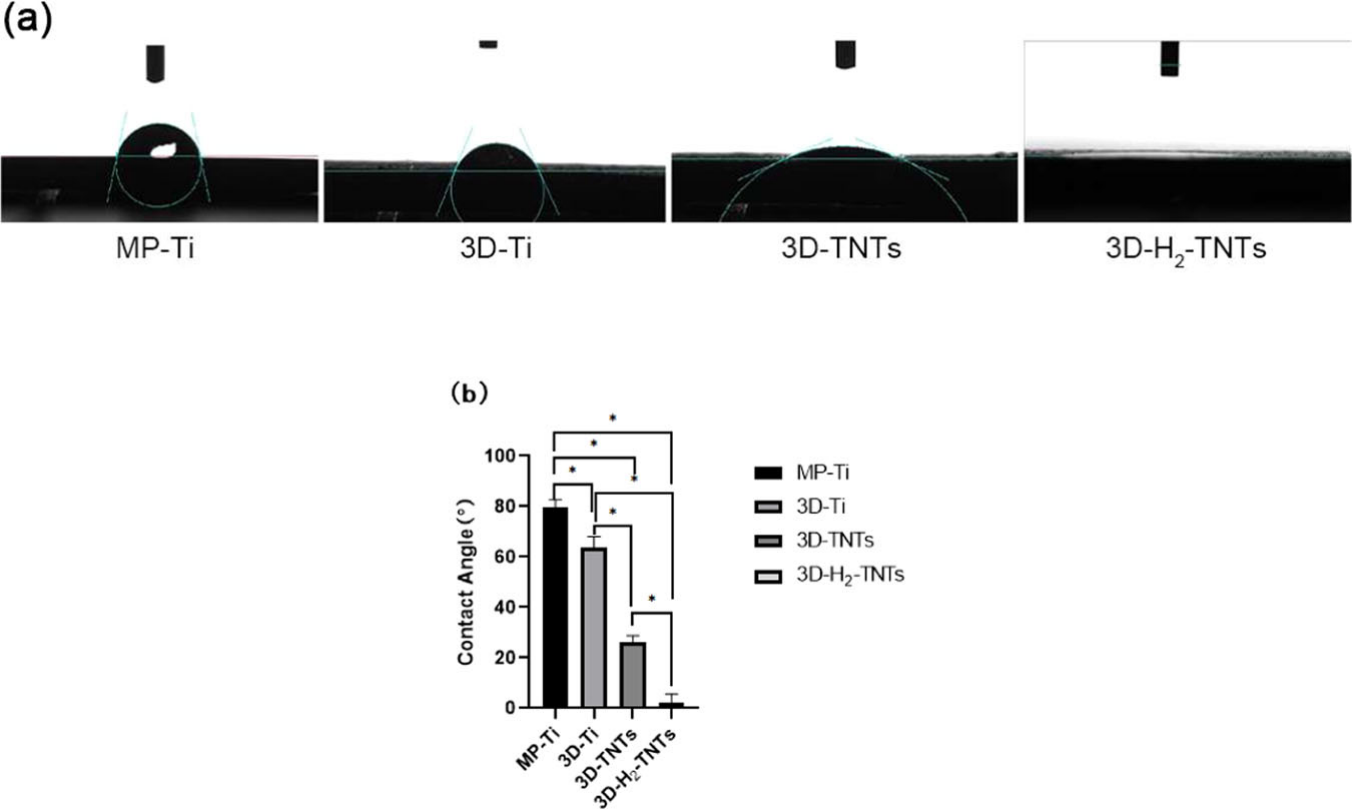
**a** Digital pictures of water contact angles for the MP-Ti, 3D-Ti, 3D-TNTs and 3D-H2-TNTs at high magnification (×30,000). **b** Water contact angles of the samples (*p < 0.05)

The water contact angles of the four groups of samples are shown in Fig. 3 and Table 1. The average water contact angle of each group was significantly different ( *p* < 0.05). The contact angles of MP-Ti and 3D-Ti were 79.76° and 63.79°, respectively. The hydrophilicity of the 3D-TNTs increased significantly, and the water contact angle decreased to 26.02° after anodizing. After further hydrogenation treatment, 3D-H_2_-TNTs obtained a superhydrophilic surface with a contact angle close to zero.

**Table 1:** Contact angle and surface roughness of the samples

Group	CA (°)	Roughness (μm)
MP-Ti	79.76 ± 2.84	0.01 ± 0.00
3D-Ti	63.79 ± 4.25^a^	6.57 ± 0.07^d^
3D-TNTs	26.02 ± 2.81^a,b^	6.66 ± 0.07^d^
3D-H_2_-TNTs	2.48 ± 0.31^a,b,c^	6.55 ± 0.06^d^

^a^Significance difference of contact angle versus MP-Ti, *p* < 0.05

^b^Significance difference of contact angle versus 3D-Ti, *p* < 0.05

^c^Significance difference of contact angle versus 3D-TNTs, *p* < 0.05

^d^Significance difference of roughness versus MP-Ti, *p* < 0.05

The surface roughness of the four groups of samples is shown in Table 1. The Ra value of MP-Ti is approximately 0.01 μm, and the roughness of 3D-Ti, 3D-TNTs and 3D-H_2_-TNTs is significantly higher than the roughness of MP-Ti with Ra value of 6.57 μm, 6.66 μm and 6.55 μm, respectively ( *p* < 0.05), but there was no statistically significant difference between the three SLM titanium alloy groups samples ( *p* > 0.05).

### Cell morphology

**Fig. 4:**
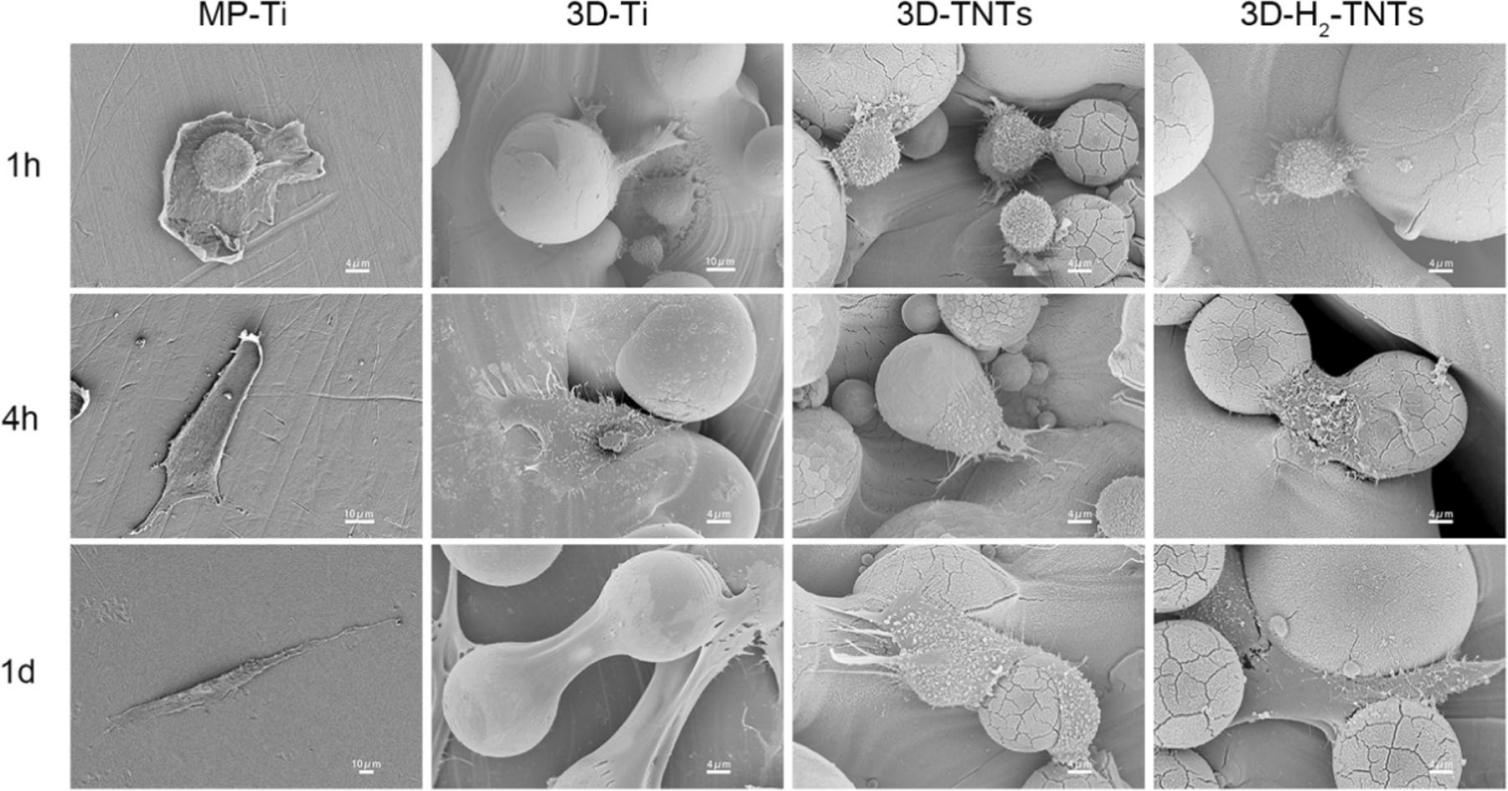
Cell morphology. SEM morphology of the HGFs adhered on the MP-Ti, 3D-Ti, 3D-TNTs and 3D-H2-TNTs after incubation for 1 h, 4 h, and 1 day

SEM photographs of HGFs cultured for 1 h, 4 h and 1 day on the four groups of samples are shown in Fig. 4. At 1 h, the cells were globose on the surface of each sample, and the nuclear contour could be seen. At the same time there were some short filopodias that could be seen protruding from the HGFs adhered on the titanium spheres of the 3D-Ti, 3D-TNTs and 3D-H_2_-TNTs, but no such phenomenon was observed on MP-Ti. Four hours after cultivation, HGFs showed a flat spindle shape on the MP-Ti, while the shape was more spread on the 3D-Ti, 3D-TNTs and 3D-H_2_-TNTs than the shape at 1 h, and the adhesion area on titanium spheres was increased. The nuclear contours of the four groups samples all disappeared. At high magnification, the pseudopods on 3D-Ti and 3D-H_2_-TNTs showed patch-like elongation and increased in number. After culturing for 1 day, HGFs were more spread spindle-shaped on MP-Ti; moreover, they were completely spread out on 3D-Ti and 3D-H_2_-TNTs and tightly attached to the titanium spheres. However, there was no marked change in cell morphology on the surface of 3D-TNTs compared with that at 4 h.

### Cell adhesion and proliferation assays

**Fig. 5:**
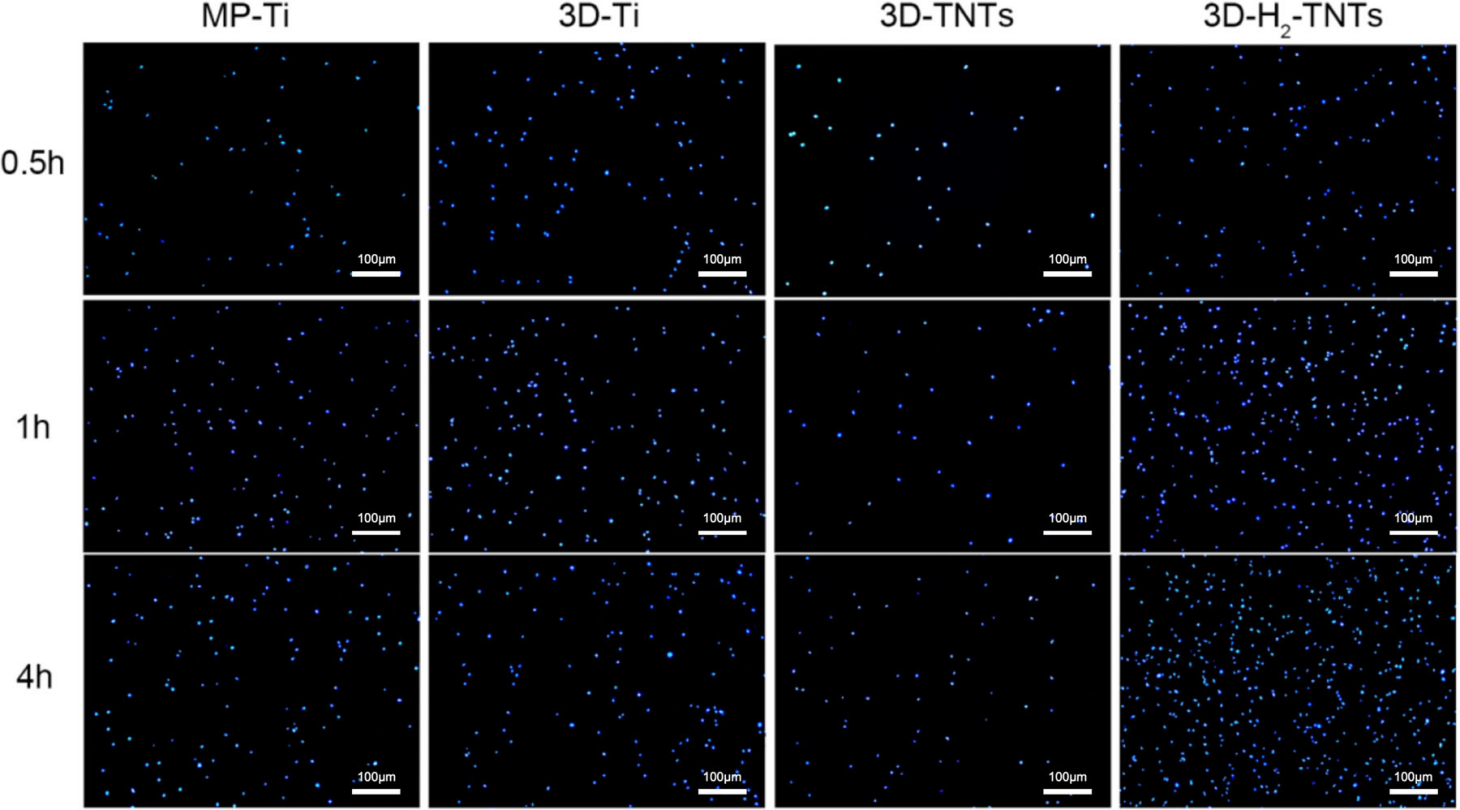
Nuclei observation with DAPI fluorescence staining. The HGFs were cultured on the MP-Ti, 3D-Ti, 3D-TNTs and 3D-H_2_-TNTs for 0.5 h, 1 h, and 4 h. The cells were labelled for nuclei (blue)

**Fig. 6:**
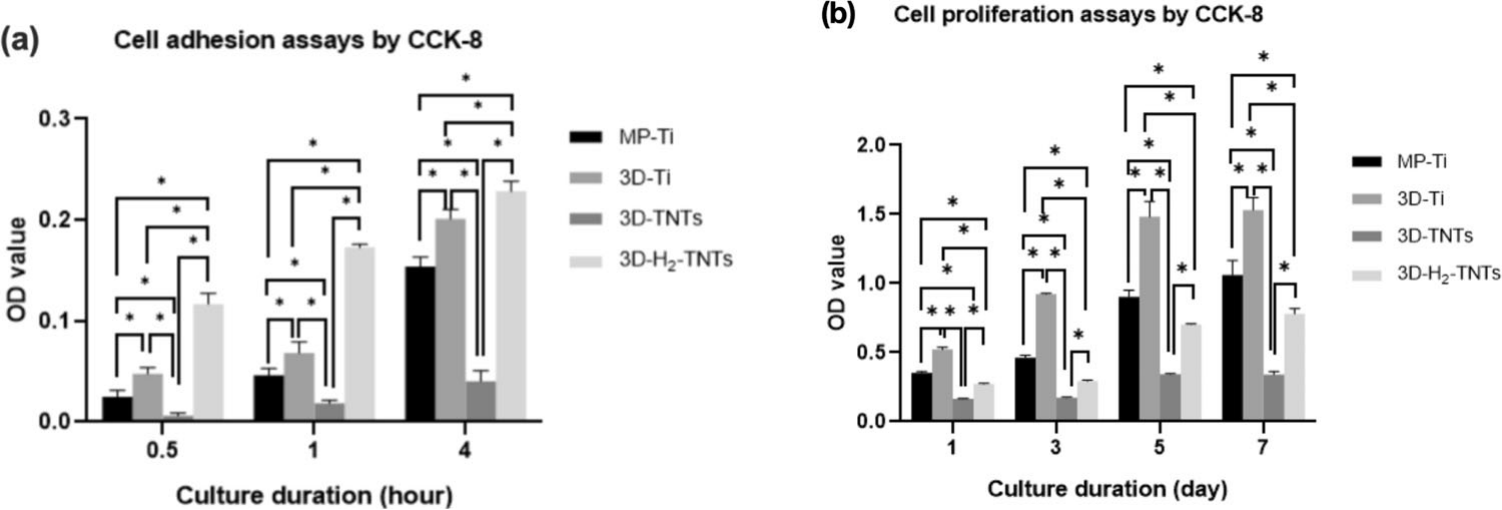
Cell adhesion and proliferation assays by CCK-8. **a** HGFs adhesion assays on the samples after incubation for 0.5 h, 1 h and 4 h (*p < 0.05 when compared with the other groups). **b** HGFs proliferation assays on the samples after incubation for 1 day, 3 days, 5 days and 7 days (*p < 0.05 when compared with the other groups)

The images of DAPI-stained cell nuclei of HGFs cultured on the samples for 0.5 h, 1 h and 4 h used for the early adhesion condition observations are shown in Fig. 5. The number of nuclei on 3D-TNTs was the lowest, while the number of nuclei on 3D-H_2_-TNTs was the highest, followed by 3D-Ti and MP-Ti. Moreover, the number of nuclei adhered to the samples in each group increased with increasing culture time. The quantitative determination of early HGF adhesion results by CCK-8 is shown in Fig. 6(a) After 0.5 h, 1 h and 4 h of culture, there were significantly more cells adhered to 3D-H_2_-TNTs than to the other three groups ( *p* < 0.05), which was consistent with the DAPI fluorescence staining results.

The proliferation of HGFs cultured on the four groups of samples was evaluated by CCK-8 assays. As indicated in Fig. 6(b), compared to the early adhesion stage, the advantage of 3D-H_2_-TNTs on cell growth disappeared when cultured for 1 day, while the cells on 3D-Ti reached the maximum at this time. We extended the culture time to 3 days, 5 days and 7 days and found that the proliferation trend of cells in each group was the same, which showed that the number of cells interacting with the surface of 3D-Ti, MP-Ti, 3D-H_2_-TNTs and 3D-TNTs decreased in turn. The proliferative activity of HGFs in all groups was found to be time-dependent. There were significant differences in proliferation between each group ( *p* < 0.05).

### Fibronectin synthesis by ELISA

**Fig. 7:**
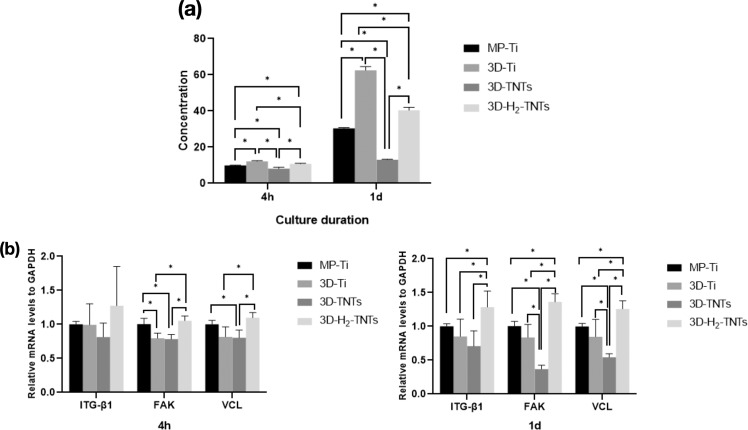
ELISA and RT–qPCR results. **a** Concentration of FN synthesized by HGFs on the four groups samples after 4 h and 1 day. **b** RT–qPCR detection of the adhesion-related gene expression of HGFs cultured on the four groups of samples for 4 h and 1 day with statistical significance by *p < 0.05. Expression of ITG-β1, FAK and VCL was presented by the relative amount of mRNA with the Formula 2^(−△△Ct)^

The concentration of fibronectin secreted by HGFs cultured on each group of samples for 4 h and 1 day measured by ELISA is shown in Fig. 7(a) The concentrations decreased in the 3D-Ti, 3D-H_2_-TNTs, MP-Ti and 3D-TNTs and increased significantly with prolonged culture time in each group. The data of each group showed a statistically significant difference ( *p* < 0.05).

### Gene expression by RT–qPCR

The expression levels of adhesion-related protein genes and extracellular matrix protein genes were analysed by RT–qPCR, as shown in Fig. 7(b). After culturing for 4 h, the mRNA expression levels of FAK, ITG-β1 and VCl in the 3D-H_2_-TNTs group were higher than the mRNA expression levels in the 3D-Ti and 3D-TNTs groups ( *p* < 0.05), but there was no statistically significant difference compared with MP-Ti ( *p* > 0.05). After culturing for 1 day, the expression of adhesion-related genes in the 3D-H_2_-TNTs group had distinct advantages and was significantly higher than the the expression of adhesion-related genes in the other three groups ( *p* < 0.05).

### Material toxicity test

**Fig. 8:**
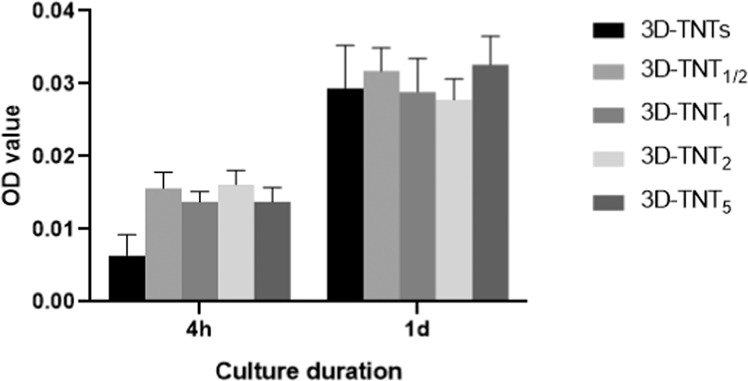
CCK-8 assays for detecting the material toxicity to HGFs. HGFs growth assays on the 3D-TNTs, 3D-TNT_1/2_, 3D-TNT_1_, 3D-TNT_2_ and 3D-TNT_5_ after incubation for 4 h and 1day

The cytotoxicity of the material analysed by CCK-8 assay is shown in Fig. 8. At both 4 h and 1 day, there was no significant difference in the absorbance between each group ( *p* > 0.05).

### Intracellular reactive oxygen species detection

**Fig. 9:**
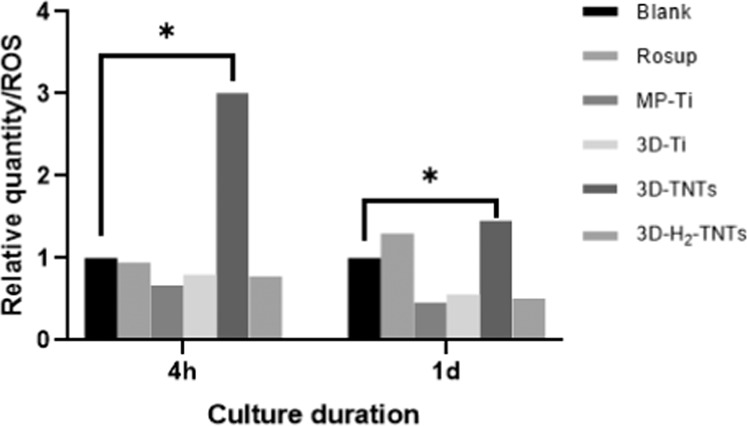
Intracellular ROS. Flow cytometry detection of the intracellular ROS of HGFs cultured on the four groups of samples for 4 h and 1 day with statistical significance at *p < 0.05

The fluorescence intensity of intracellular ROS analysed by flow cytometry is shown in Fig. 9. At 4 h and 1 day, the fluorescence intensity of intracellular ROS in the 3D-TNTs was 10^5^ and 10^6^, respectively, which were significantly higher than the the fluorescence intensity of intracellular ROS in the blank group, which were 10^4^ and 10^5^, respectively, and the other three test groups ( *p* < 0.05). There was no significant difference between the other three groups compared with the blank group ( *p* > 0.05).

## Discussion

In this study, a superhydrophilic microsphere-nanotube composite morphology was constructed on the surface of SLM titanium alloy by electrochemical anodic oxidation and high-temperature hydrogenation, and HGFs were cultured on the surface of the material. Early cell adhesion, proliferation, adhesion-related genes and protein expression were detected to evaluate the HGFs biocompatibility of the material.

The surface morphology, roughness and hydrophilicity of materials are important factors affecting the biological behaviour of cells [14]. Research shows that TiO_2_ nanotubes 100 nm in diameter are easily identified and connected by plasma fibronectin of similar size. Then, plasma fibronectin transmits the signals of surface topography to HGFs and launches a series of signalling pathways, which can help HGFs bind to HGFs with the aid of transmembrane proteins and integrins [15–17]. Compared with smooth titanium, a rough titanium surface with a micro-nano composite morphology is more conducive to the adhesion of HGFs [18–22]. Many experiments applied different technologies, such as laser etching and large-particle sandblasting acid etching, to modify the surface of implant material, which could efficiently improve the hydrophilicity of the material. Some studies indicated that the hydrophilic interface can enhance the expression of FN and collagen fibre by promoting both the proteins and other biological macromolecules adhering to the material and the cellular interactions with the adhesive layer to promote the adhesion of HGFs and form a stronger combination with the material [23–25].

In this experiment, 3D-Ti was prepared by SLM technology. By reason of the spheroidization of titanium alloy powder in the melting process and we did not carry out any polishing treatment after the material generated, as shown in Fig. 2(B)/(b), there were a large number of raised titanium spheres on the surface of 3D-Ti, and the diameter of most of the titanium spheres was approximately 30 μm. At this point, the surface water contact angle of the material is approximately 64° (Fig. 3, Table 1), which is significantly reduced compared with MP-Ti. Then, 3D-Ti was electrochemically anodized, and TiO_2_ nanotubes with diameters of 80–100 nm were prepared on the surface of titanium spheres (Fig. 22(C)/(c)). 3D-TNTs obtained a micro-nano composite morphology, and the surface hydrophilicity further increased. On this basis, hydrophilic 3D-H_2_-TNTs were obtained by high-temperature hydrogenation. The preliminary studies of our research group confirmed that high-temperature hydrogenation treatment can generate more oxygen vacancies and T^3+^ on the surface of TiO_2_ and can react with H_2_O to generate hydrophilic domain -OH to significantly improve the hydrophilicity of the material [26].

Next, we tested the biological behaviour of the HGFs. SEM and CCK-8 results (Fig. 4, Fig. 6(a)) showed that in the early 4 h of culture, some short pseudopodia of cells could be observed in contact with titanium spheres and nanotubes on the surface of three SLM titanium alloy sample groups, and the number of adhered HGFs on 3D-H_2_-TNTs was significantly greater than that of the other three groups. Early adhesion between cells and the material surface is the basis for later migration, differentiation and proliferation [27]; thus, good adhesion of HGFs on 3D-H_2_-TNTs within 4 h is of great significance for later proliferation and the expression of adhesion-related protein molecules and genes. The adhesion and proliferation of cells are closely related to the interaction of cells with extracellular matrix proteins and transmembrane proteins on the surface of the material [28]. Fibroblasts can secrete fibronectin (FN) and attach to the surface of materials in the form of focal adhesion (FAs) with the help of transmembrane proteins and integrin [29]. We speculated that compared with smooth titanium, the microsphere-nanotube structure on the surface of SLM titanium alloy samples provided a bionic environment that was similar to the extracellular matrix for HGFs, and the FAs were more likely to form attachment sites on these structures to promote cell colonization [30–32]. With the extension of culture time to 1 day, HGFs on 3D-Ti and 3D-H_2_-TNTs were completely spread out and tightly wrapped on the spheres and nanotubes; however, the number of cells in the 3D-Ti group was the highest (Fig. 6(a)). We speculated that contact inhibition may have occurred in the 3D-H_2_-TNTs group due to too many cells adhering to the sample in the early stage and the cell state being degraded with the consumption of nutrients and the accumulation of metabolites in the medium.

FAs are a complex collection of plasma membrane-related macromolecules that are mainly composed of the transmembrane protein ITG-β1, adherent plaque kinase FAK and intracellular skeleton binding protein VCL [33–35]. RT–qPCR results (Fig. 7(b)) showed that the gene expression levels of ITG-β1, FAK and VCL in the 3D-H_2_-TNTs group were significantly higher than the gene expression levels in the other three groups at 4 h and 1 day. We hypothesized that the surface microenvironment of 3D-H_2_-TNTs may induce the adhesion of a large number of HGFs, leading to an increasing number of pseudopodia stretching out from the cells to anchor stably on the 3D-H_2_-TNTs sample and guiding the HGFs to secrete dense extracellular matrix proteins. Meanwhile, it could also improve the expression level of the genes related to cell transmembrane proteins to produce more anchor points and promote the maturation of FAs and more cell adhesion. Ultimately, the secretion and deposition of extracellular matrix are improved, which may be the main reason for the good biological behaviour of HGFs on 3D-H_2_-TNTs. Our ELISA results shown in Fig. 7(a) indicated that the secretion volume of FN in the 3D-H_2_-TNTs group was significantly higher than the secretion volume of FN in the MP-Ti group at 4 h but lower than the secretion volume of FN in the 3D-Ti group. With the extension of culture time to 1 day, FN secretion on the surface of each group increased, but the FN secretion on the surface of 3D-H_2_-TNTs was still lower than the FN secretion on the surface of 3D-Ti. As a secreted protein, the synthesis and secretion process of FN involves a variety of organelles and is affected by cell signals. We speculate that the too complicated surface morphology of 3D-H_2_-TNTs compared with 3D-Ti affects the intracellular signal transduction of HGFs, thus weakening the expression and secretion of FN, but the specific mechanism is not yet clear.

We found that the 3D-TNTs group had the fewest adhered cells and the lowest expression of adhesion-related proteins and genes in both early adhesion and later proliferation stages (Fig. 6, Fig. 7). We speculated that the negative results of the 3D-TNTs group in this experiment may be due to the cytotoxicity of the electrolyte remaining in the nanotubes after anodic oxidation. On the other hand, this may be due to the too complicated micro-nano morphology of the material, which initiated a series of signalling pathways that inhibit cell growth. Previously, we looked at the relevant literatures and found that although many studies on nanotubes showed that they could significantly promote the growth of HGFs, some studies proposed that nanostructures could inhibit the proliferation and extension of cells in terms of spatial orientation and structural arrangement [36, 37], and excessive intracellular ROS can initiate the apoptosis process of cells [38]. Based on the above hypotheses, we explored the two reasons that may lead to the negative results of the 3D-TNTs group. To verify the first assumption, we cleaned the 3D-Ti those after anodizing with different ultrasonication times. SEM and CCK-8 results showed that only some nanotubes of 3D-TNT_5_ with ultrasonic cleaning for 5 min had irregular fracture, while the morphology of the other groups with different ultrasonic cleaning times did not change compared with 3D-TNTs. Moreover, the absorbance of HGFs cultured on several groups of materials for 4 h and 1 day showed no significant difference (Fig. 8), so we excluded the first hypothesis. To verify the second hypothesis, we tested the concentration of intracellular ROS, and the results (Fig. 9) showed that the concentration of 3D-TNTs was significantly higher than the concentration of 3D-TNTs in the negative control group and the other three groups at both 4 h and 1 day. Therefore, we speculated that in addition to transmitting spatial signals to HGFs, the too complicated morphology of 3D-TNTs also activated a variety of negative signalling pathways to inhibit cell activity by changing the cell morphology [39], which adversely affected cell growth. However, the specific mechanism remains to be further explored. At the same time, the superhydrophilicity of 3D-H_2_-TNTs compensated for such inhibition in the early stage, so 3D-H_2_-TNTs showed the best biocompatibility for cells. As time went on, the advantage of hydrogenation disappeared, and the 3D-Ti without any treatment showed superiority in the proliferation of HGFs.

In conclusion, the superhydrophilic micro-nano composite morphology of 3D-H_2_-TNTs can significantly promote the early adhesion and proliferation of HGFs and increase the secretion of fibronectin and the expression of FA-related genes, which suggests that the application of the material to the transgingival area of the implant has the potential to produce rapid and firm connective tissue attachment and good soft tissue healing in the early stage. However, further animal experiments are needed to verify the effect of 3D-H_2_-TNTs on HGFs. Meanwhile, we also need to consider how to remove the negative effect of TiO_2_ nanotubes on cells to sustain the superior effect of modified materials on cells.
